# The association between high-sensitivity C-reactive protein and blood pressure in Yi people

**DOI:** 10.1186/s12889-019-7324-x

**Published:** 2019-07-24

**Authors:** Li Pan, Guoju Li, Shaoping Wan, WuLi Yihuo, Fang Yang, Zheng Li, Guangliang Shan

**Affiliations:** 1 Department of Epidemiology and Statistics, Institute of Basic Medical Sciences, Chinese Academy of Medical Sciences, School of Basic Medicine, Peking Union Medical College, Beijing, 100005 China; 2 Qingdao Women and Children’s Hospital, Qingdao University, Qingdao, 266011 Shandong China; 3 Sichuan Provincial Hospital, Chengdu, China; 4 Department for Chronic Noncommunicable Diseases Control, Puge County Center for Disease Control and Prevention, Xichang, Sichuan China; 5 Xichang Municipal Center for Disease Control and Prevention, Xichang, Sichuan China

**Keywords:** Blood pressure, High-sensitivity C-reactive protein (hs-CRP), Yi people

## Abstract

**Background:**

High-sensitivity C-reactive protein (hs-CRP) is a common risk factor for developing cardiovascular disease. However, there has been no study reporting the relationship between hs-CRP and blood pressure in Yi adults. The aim of this study is to investigate the association between hs-CRP and blood pressure in Yi adults.

**Methods:**

In this cross-sectional study, included subjects were 2916 Yi migrants or farmers aged 20–80 years, recruited by using a stratified cluster sampling method from Liangshan Yi Autonomous Prefecture of Sichuan Province in 2014. The directed acyclic graphs(DAG) was used to select a minimal sufficient adjustment sets of variables which would identification the unconfounded effect of hs-CRP and hypertension. Multiple linear and multinomial logit analysis were used to estimate the effect of hs-CRP on SBP/DBP/MAP/PP and the prevalence of prehypertension/hypertension after adjustment for the relevant confounders.

**Results:**

The median level of hs-CRP was 1.20 (0.50–3.06)mg/L in Yi migrants, and 0.84(0.36–2.52) mg/L in Yi farmers, and the prevalence of high hs-CRP was 23.25%. For hs-CRP > 3 mg/L group, the adjusted PP tended to have lower values (β = − 1.49, 95%CI: − 2.49--0.49, *P* = 0.0034) compared with < 1 mg/L group. After adjusting for confounders, there were no significant association between hs-CRP and prehypertension/hypertension (*P* > 0.05).

**Conclusions:**

Our results suggest that high hs-CRP is prevalent in Yi people, and this study does not support hs-CRP as a risk factor of prehypertension or hypertension.

## Background

As a primary risk factor of cardiovascular disease [1], hypertension has affected 23.2% of the Chinese adult population (approximately 244.5 million) with the age ≥ 18 years old [2]. Among various Blood Pressure (BP) indices, Systolic Blood Pressure (SBP), Diastolic Blood Pressure (DBP), Mean Arterial Pressure (MAP), and Pulse Pressure (PP) have been broadly applied [3]. It has been reported that even the slightly elevated blood pressure within the normal range may lead to cardiovascular morbidity and mortality [4, 5] [6]. As a pentagonal acute phase reactant molecule, high-sensitivity C-Reactive Protein (hs-CRP) was initially produced in hepatocytes stimulated by interleukin-6 (IL-6) and Tumor Necrosis Factor (TNF), which were the biomarkers of systemic inflammation [7]. Hs-CRP was demonstrated to serve as an indicator to identify the risk of cardiovascular events in patients with high blood pressure [8–10]. Additionally, a meta-analysis of cohort studies revealed that high levels of hs-CRP was associated with the risk of hypertension development [11]. Although hs-CRP may be an early marker of hypertension, there is little evidence that confirmed the association between the levels of hs-CRP and BP indices. Besides, a variety of studies have indicated the inhomogeneity of the levels of hs-CRP among different ethnicities or races [12–14].

Ethnicity or race has been considered as the determining factor in the expression levels of biomarkers and played a crucial role in the relationship with cardiovascular diseases [15]. Located in the southwest of Sichuan Province, China, Liangshan Yi Autonomous Prefecture consists of 17 counties (cities) and 48.9% of Yi ethnic people, which is the largest Yi community in China [16]. Due to the isolation from the outside world, the farmers in Yi population have maintained the less acculturated lifestyle. In contrast to the farmers, the migrants in Yi population and Han residents who lived in the same communities with the similar diets composed of rice, meat and fresh vegetables, have experienced a evolution from the traditional lifestyle to the more typical urbanized one with higher fat intake [17]. Several researches have investigated hs-CRP in Yi people and the data revealed that there was no correlation between hs-CRP and BPs. Therefore, the objective of this study was to explore the relationship between hs-CRP and BPs in the adults of Yi population.

## Methods

### Study population

This Study was conducted from April 2015 to November 2015 in Liangshan Yi Autonomous Prefecture, Sichuan Province. The sampling procedure has been published in detail [18]. By using stratified cluster sampling method, the Yi people aged 20 to 80 years were selected as the subjects of the survey. The Liangshan Yi Autonomous Prefecture was stratified into urban and rural areas, and Xichang city was selected from urban areas and Puge County was selected from rural areas by using the convenience sampling. In each area, different communities or townships were treated as sampling units and three communities from Xichang City, five rural townships from Puge County were randomly selected. All Yi farmers in selected villages were surveyed. The Yi migrants were Yi people who had migrated to a county or Xichang city for over 1 year prior to the survey. The survey was approved by the Institutional Review Board of the Institute of Basic Medical Sciences, Chinese Academy of Medical Sciences, and informed consent was obtained from all participants.

### Health survey

Demographic information, smoking habit, alcohol drinking habit and history of diseases were collected by unified questionnaires. Ethnicity was determined based on the subjects’ ID card. Yi people were defined as the individuals and they and their parents were all Yi people. Subjects were classified as smokers (smoke more than one cigarette per day and last more than 6 months), former smoker and never smoker. For alcohol consumption, subjects were classified as current drinker (ever drinking), former drinker, and never drinker.

### Measurements

Height and weight were measured with no shoes and light clothing by trained research assistants using a measurement device (BC-420, TANITA). Body Mass Index (BMI) was calculated as the weight in kilograms divided by the square of height in meters. Sitting blood pressure were collected triply by experienced physicians based on the HEM-907 equipment on the right arm positioned at heart level after a rest for 10 min and the subjects were not allowed to smoke, exercise or feed. Three independent measurements were averaged for all analyses.

After overnight fasting (at least 8 h), blood samples were taken and processed immediately. And then they were refrigerated, transported to Beijing laboratory and stored below minus 80 °C before analysis. Total cholesterol (TC), triglycerides (TG), high-density lipoprotein cholesterol (HDL-C), low-density lipoprotein cholesterol (LDL-C), and hs-CRP was measured by immunoturbidimetry. Hepingli Hospital laboratory passed the quality control test conducted by National Center for Clinical Laboratories, where daily dual-level indoor quality control was taken.

### Definitions

Hs-CRP was classified according to the recommendation of the American Heart Association and Center for Disease Control, defining hs-CRP < 1 mg/L as low risk, between 1 and 3 mg/L as moderate risk, and > 3 mg/L as high risk [19].

BMI was divided into three groups, including thin or healthy (BMI < 25 kg/m^2^), overweight (25 < BMI < 29 kg/m^2^) and obese (BMI ≥ 30 kg/m^2^) groups based on the criteria made by World Health Organization [20]. Dyslipidemia was defined as Total Cholesterol (TC) ≥ 6.22 mmol/L and/or triglyceride (TG) ≥ 2.26 mmol/L and/or High Density Lipoprotein Cholesterol (HDL-C) < 1.04 mmol/L and/or Low Density Lipoprotein Cholesterol (LDL-C) ≥ 4.14 mmol/L [21]. Three clusters including optimal blood pressure, prehypertension, and hypertension, optimal blood pressure were grouped on account of blood pressure. Specifically, the average SBP < 120 mmHg and DBP < 80 mmHg were considered as optimal blood pressure, and the average SBP ranging from 120 to 139 mmHg and/or DBP 80–89 mmHg as prehypertension [22]. Besides, hypertension was reported as the mean SBP ≥ 140 mmHg, and/or DBP ≥ 90 mmHg, or self-reported diagnosis of hypertension [23].

### Statistical analysis

**Fig. 1:**
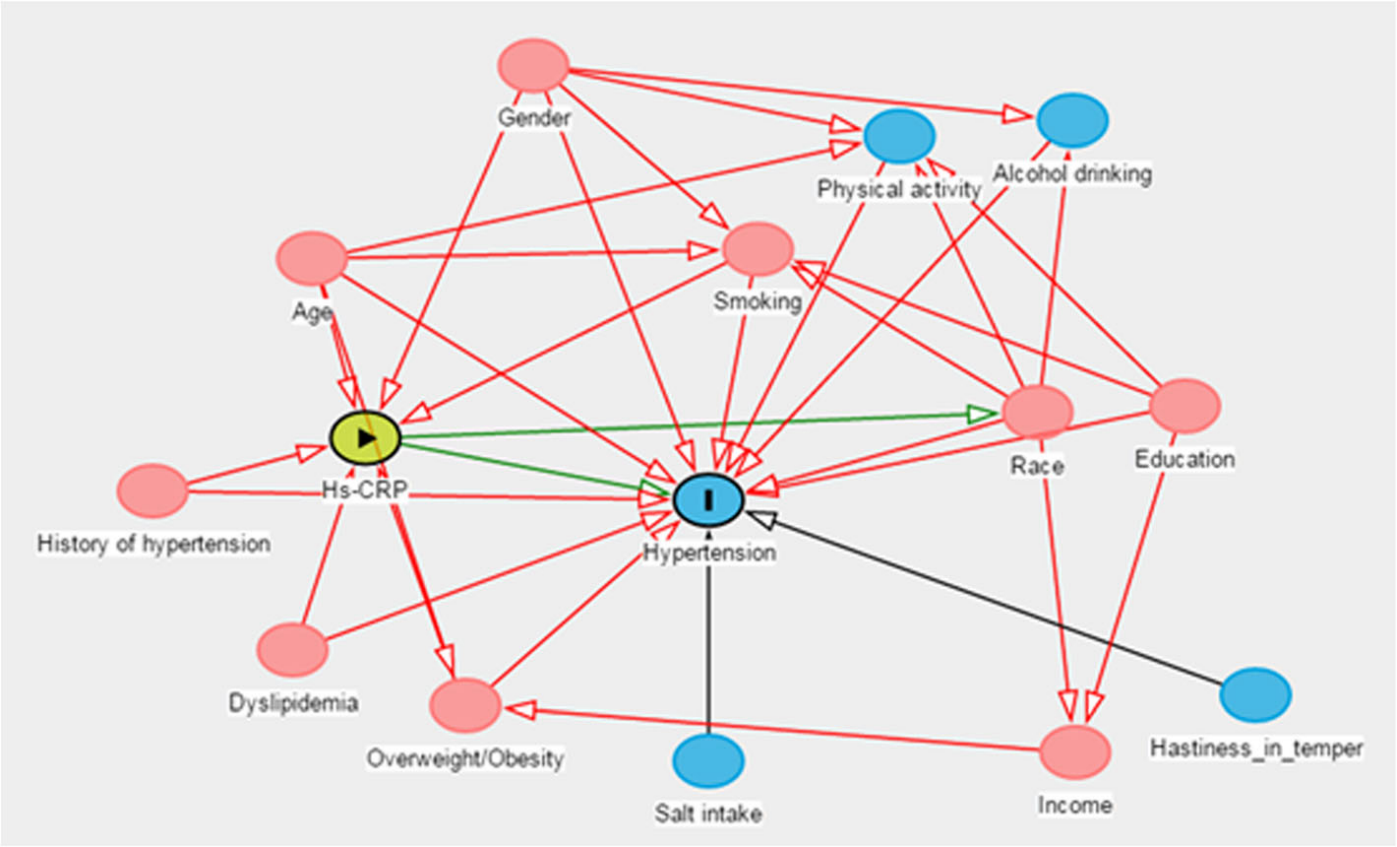
Directed acyclic graph (DAG) for the confounded effect of hs-CRP and hypertension

About 11% of the individuals were excluded from the analysis due to missing data on hs-CRP or other variable and excluding these adults would reduce statistical power and increased the likelihood of residual confounding. So we used multivariate imputation(MI) to fill out the missing values [24]. The directed acyclic graphs(DAG) was used to select a minimal sufficient adjustment sets of variables which would identification the unconfounded effect of hs-CRP and blood pressure, and the DAG was implemented by DAGitty [25, 26]. The DAG was implemented by identifying the main risk factors affecting hs-CRP or hypertension. We finally included gender, age, education, history of hypertension, smoking, overweight/obesity and dyslipidemia (Fig. 1). The Yi farmers or Yi migrants were the main factor of this study, so we also included it. Excluding individuals receiving pharmacological treatment for hypertension may have resulted in selection bias if the decision to treat was influenced by other conditions associated with hs-CRP levels, and to address this issue, we included treatment as a factor in the analysis.

Continuous variables were described as mean ± SD, and categorical variables were expressed as numbers and percentages. For skewed variables (triglyceride levels, hs-CRP), median (inter-quartile range) were used. MAP was defined as DBP plus 1/3 PP, and PP was defined as SBP minus DBP. Trend tests across different levels of hs-CRP for continuous and categorical variables were conducted using general linear model and Cochran-Armitage test, correspondingly. With the increase of hs-CRP, the increasing trends in mean systolic blood pressure (SBP), diastolic blood pressure (DBP), mean arterial pressure (MAP), and pulse pressure (PP) values were tested by the analysis of covariance.

Multiple linear and multinomial logit analysis were used to estimate the effect of hs-CRP on SBP/DBP/MAP/PP and the prevalence of prehypertension/hypertension after adjustment for gender, age, Yi farmers or Yi migrants, education, history of hypertension, smoking, overweight/obesity and dyslipidemia.

We also evaluated the interaction between age and gender, age and overweight/obesity interaction terms in the regression models. The age-squared was included in the regression models to account for the non-linearity. This way to select model factors and functional forms avoided the bias. Hosmer and Lemeshow test was used to test the fit of the Logistic model, Studentized residual and Cook’s distance were used to test the fit of the linear models.

## Results

The analysis included 2916 individuals, of which 1204(41.29%) were Yi migrants. The average age was 47.77 ± 14.33 years in Yi migrants, and 45.16 ± 12.74 years in Yi farmers. Compared with the Yi farmers, the Yi migrants had higher proportions of females, high education level, alcohol drinkers, family history of hypertension, and were more likely to have a higher BMI, TC, TG, LDL-C, SBP, DBP, MAP and hs-CRP (all *P* < 0.05). Yi farmers appeared to have a higher HDL-C compared with Yi migrants (*P* < 0.05). The median of hs-CRP level was 1.20 (0.50–3.06)mg/L in Yi migrants, and 0.84(0.36–2.52) mg/L in Yi farmers(Table 1).

**Table 1:** Characteristics of the study population according to location

Characteristics	Yi migrants (*N* = 1204)	Yi farmers (*N* = 1712)	*P* value
Male, %	373(30.98)	577(33.70)	0.12
Age, mean(SD), y	47.77 ± 14.33	45.16 ± 12.74	**< 0.0001**
BMI, mean(SD), kg/m^2^	24.27 ± 3.84	22.11 ± 3.55	**< 0.0001**
Cigarette Smokers, %	365(30.32)	562(32.83)	0.15
Alcohol drinkers, %	513(42.71)	647(37.84)	**0.0081**
Family history of hypertension, %	120(9.97)	57(3.33)	**< 0.0001**
Education Level > elementary, %	586(48.67)	194(11.33)	**< 0.0001**
Total cholesterol, mmol/L	5.08 ± 1.01	4.96 ± 1.01	**0.0034**
Triglycerides, mmol/L	1.41(1.01–2.02)	1.12(0.85–1.61)	**< 0.0001**
LDL cholesterol, mmol/L	3.17 ± 0.90	3.06 ± 0.86	**0.0008**
HDL cholesterol, mmol/L	1.18 ± 0.29	1.24 ± 0.30	**< 0.0001**
Dyslipidemia,%	568(47.18)	641(37.44)	**< 0.0001**
Systolic blood pressure, mmHg	121.40 ± 18.84	118.40 ± 16.45	**< 0.0001**
Diastolic blood pressure, mmHg	74.92 ± 11.14	72.07 ± 10.95	**< 0.0001**
Pluse pressure, mmHg	46.46 ± 12.82	46.30 ± 10.82	0.72
Mean arterial pressure, mmHg	90.41 ± 12.83	87.50 ± 12.00	**< 0.0001**
C-Reactive Protein, mg/L	1.20(0.50–3.06)	0.84(0.36–2.52)	**< 0.0001**

Data are presented as means ± SD or *n* (%) or median (Interquartile range)

The symbol "bold" reflected the *P* < 0.05

The characteristics of the subjects stratified by the levels of C-reactive protein for Yi farmers and Yi migrants are shown in Table 2. Mean age, BMI, TC, TG, LDL cholesterol, SBP, DBP, MAP, the proportion of smokers, alcohol drinkers and dyslipidemia increased with hs-CRP (all *P* for trend < 0.05), while the mean level of HDL cholesterol decreased (*P* for trend < 0.001) in both Yi farmers and Yi migrants. Mean PP increased with hs-CRP (*P* for trend< 0.001) in Yi migrants.

**Table 2:** Comparison of characteristics among 2716 Yi people according to level of C-reactive protein among Yi farmers and Yi migrants

Characteristics	Level of C-Reactive Protein, mg/L			P for Trend
	< 1	1~3	> 3	
Yi migrants				
Number	529	369	306	
C-Reactive Protein, median(interquartile range), mg/L	0.43(0.26–0.67)	1.64(1.27–2.19)	5.92(4.32–11.09)	
Age, mean(SD), y	43.22 ± 13.21	50.08 ± 14.10	52.87 ± 14.12	**< 0.0001**
BMI, mean(SD), kg/m^2^	23.20 ± 3.26	24.97 ± 3.73	25.27 ± 4.39	**< 0.0001**
Cigarette Smokers, %	136(25.71)	116(31.44)	113(36.93)	**0.0006**
Alcohol drinkers, %	204(38.56)	169(45.92)	140(46.05)	**0.021**
Education Level > elementary, %	264(49.91)	190(51.49)	132(43.14)	0.1
Family history of hypertension, %	54(10.21)	36(9.76)	30(9.80)	0.83
Total cholesterol, mmol/L	4.93 ± 0.99	5.15 ± 0.99	5.25 ± 1.06	**< 0.0001**
Triglycerides, mmol/L	1.22(0.91–1.75)	1.53(1.11–2.17)	1.61(1.16–2.21)	**< 0.0001**
LDL cholesterol, mmol/L	3.04 ± 0.88	3.24 ± 0.85	3.31 ± 0.95	**< 0.0001**
HDL cholesterol, mmol/L	1.24 ± 0.30	1.14 ± 0.27	1.10 ± 0.27	**< 0.0001**
Dyslipidemia,%	184(34.78)	198(53.66)	186(60.78)	**< 0.0001**
Systolic blood pressure, mmHg	117.47 ± 17.76	124.28 ± 19.22	124.66 ± 18.99	**< 0.0001**
Diastolic blood pressure, mmHg	73.18 ± 11.04	76.21 ± 11.06	76.38 ± 11.04	**0.0001**
Pulse pressure, mmHg	44.29 ± 11.38	48.07 ± 14.02	48.28 ± 13.11	**< 0.0001**
Mean arterial pressure, mmHg	87.94 ± 12.55	92.23 ± 12.69	92.48 ± 12.78	**< 0.0001**
Yi farmers				
Number	936	404	372	
C-Reactive Protein, median(interquartile range), mg/L	0.39(0.24–0.60)	1.70(1.35–2.23)	6.59(4.19–11.95)	
Age, mean(SD), y	43.31 ± 12.20	47.33 ± 13.04	47.47 ± 13.03	**< 0.0001**
BMI, mean(SD), kg/m^2^	21.58 ± 3.02	22.82 ± 3.76	22.65 ± 4.28	**0.0001**
Cigarette Smokers, %	259(27.67)	147(36.39)	156(41.94)	**< 0.0001**
Alcohol drinkers, %	306(32.73)	166(41.19)	175(47.04)	**< 0.0001**
Education Level > elementary, %	93(9.94)	54(13.37)	47(12.63)	0.09
Family history of hypertension, %	32(3.42)	14(3.47)	11(2.96)	0.71
Total cholesterol, mmol/L	4.89 ± 0.94	5.10 ± 0.99	5.00 ± 1.18	**0.0211**
Triglycerides, mmol/L	1.07(0.82–1.50)	1.21(0.89–1.77)	1.17(0.91–1.74)	**< 0.0001**
LDL cholesterol, mmol/L	3.00 ± 0.80	3.16 ± 0.86	3.09 ± 0.98	**0.0154**
HDL cholesterol, mmol/L	1.27 ± 0.29	1.23 ± 0.31	1.15 ± 0.31	**< 0.0001**
Dyslipidemia,%	279(29.81)	172(42.57)	190(51.08)	**< 0.0001**
Systolic blood pressure, mmHg	116.80 ± 15.75	120.48 ± 17.23	120.02 ± 16.93	**0.0002**
Diastolic blood pressure, mmHg	70.94 ± 10.49	72.55 ± 10.99	74.37 ± 11.65	**< 0.0001**
Pulse pressure, mmHg	45.86 ± 10.32	47.93 ± 11.74	45.65 ± 10.85	0.63
Mean arterial pressure, mmHg	86.23 ± 11.50	88.52 ± 12.20	89.59 ± 12.64	**< 0.0001**

Data are presented as means ± SD or *n* (%) or median (Interquartile range)

The symbol "bold" reflected the *P* < 0.05

Table 3 presents the results of association between hs-CRP levels and BP. In the unadjusted analyses, compared with the hs-CRP < 1 mg/L group, the 1-3 mg/L and > 3 mg/L groups appeared to have a higher values of SBP, DBP, PP and MAP (all *P* < 0.05). After adjusting for gender, age, age2, gender*age, Yi farmers or Yi migrants, education, family history of hypertension, smoking, overweight/obesity, age*overweight/obesity, pharmacological treatment for hypertension, and dyslipidemia, the PP (β = − 1.49, 95%CI: − 2.49--0.49, *P* = 0.0034) appeared to have lower values in hs-CRP > 3 mg/L group.

**Table 3:** Average difference in blood pressure(mmHg) by level of hs-CRP

Models	CRP Levels, mg/L	Change in SBP (*n* = 2916)	95%CI	*P-*Value	Change in DBP (*n* = 2916)	95%CI	*P-*Value	Change in PP (*n* = 2916)	95%CI	*P-*Value	Change in MAP (*n* = 2916)	95%CI	*P-*Value
Unadjusted	< 1(reference)	0.00			0.00			0.00			0.00		
	1–3	5.25	3.74, 6.76	< 0.0001	2.54	1.58, 3.50	< 0.0001	2.71	1.70, 3.72	< 0.0001	3.45	2.38–4.52	< 0.0001
	> 3	5.07	3.49, 6.65	< 0.0001	3.52	2.53, 4.53	< 0.0001	1.55	0.49, 2.60	0.0042	4.04	2.92–5.16	< 0.0001
	< 1(reference)	0.00			0.00			0.00			0.00		
	1–3	0.24	−1.13, 1.61	0.73	−0.03	−0.93, 0.88	0.95	0.27	−0.67, 1.20	0.58	0.06	−0.93-1.05	0.90
	> 3	−1.04	−2.49, 0.41	0.16	0.45	−0.51, 1.41	0.36	−1.49	**−2.48, −0.49**	**0.0034**	−0.05	− 1.10-1.00	0.93

+Adjusted: gender, age, age2, gender*age, Yi farmers or Yi migrants, education, history of hypertension, smoking, overweight/obesity, age*overweight/obesity, pharmacological treatment for hypertension, and dyslipidemia

The symbol "bold" reflected the *P* < 0.05

Table 4 presents the results of the associations between hs-CRP levels and prehypertension/hypertension. In crude models, higher prevalence of prehypertension/hypertension were associated with increasing levels of hs-CRP (all OR > 1, *P* < 0.05). Then we only adjusted gender and age and found the prevalences of pre-hypertension and hypertension were both higher in hs-CRP 1-3 mg/L group (all OR > 1, *P* < 0.05 compare with hs-CRP < 1 mg/L group), and the prevalence of hypertension was higher in hs-CRP > 3 mg/L group(OR = 1.69, 95%CI: 1.29–2.22). After multiple confounders adjustment described before, there were no significant association between hs-CRP and prehypertension/hypertension (*P* > 0.05).

**Table 4:** Association between blood pressure levels and C-reactive protein by multivariate logistic regression models

CRP Levels	Pre-hypertension Odds Ratio (95%CI) (*n* = 865)			Hypertension Odds Ratio (95%CI) (*n* = 491)		
	Unadjusted	Age and sex adjusted	Multivariate adjusted+	Unadjusted	Age and sex adjusted	Multivariate adjusted+
< 1	1	1	1	1	1	1
1–3	1.64(1.35–2.00) *	1.37(1.12–1.69) *	1.06(0.85–1.32)	2.26(1.76–2.90) *	1.57(1.20–2.05) *	1.08(0.81–1.43)
> 3	1.57(1.27–1.94) *	1.22(0.98–1.52)	0.91(0.72–1.16)	2.75(2.14–3.54) *	1.69(1.29–2.22) *	1.11(0.83–1.48)

*Compared with hs-CRP < 1: *P* < 0.05; +Adjusted: gender, age, age2, gender*age, Yi farmers or Yi migrants, education, history of hypertension, smoking, overweight/obesity, age*overweight/obesity, and dyslipidemia

The symbol "bold" reflected the *P* < 0.05

## Discussion

To the best of our knowledge, this is the first study to investigate the association between hs-CRP and BP in Yi people in Liangshan Yi Autonomous Prefecture. In this study, the prevalence of high hs-CRP was 23%, and one study reported the prevalence of high hs-CRP was approximately 25% in the general population in the United States [27]. Another study of 11623 middle-aged Chinese people reported the prevalence of hs-CRP ≥3 mg/L as 12.3%, and the median hs-CRP level of the study was 0.80 mg/L, which was similar to ours at 0.99 mg/L [28]. A meta-analysis also reported the median level of hs-CRP was 0.97 mg/L in East Asian [12]. In general, several reports pointed out Asian people had lower hs-CRP levels than western people did. Women’s Health Across the Nation (SWAN) study showed that African-American women had the highest median hs-CRP level (3.2 mg/L), followed by Hispanic (2.3 mg/L), white (1.5 mg/L), Chinese (0.7 mg/L), and Japanese (0.5 mg/L) women [29].

Another important finding was hs-CRP was positively associated with SBP, DBP, MAP and PP. However, the associations did not persist after the adjusting for relevant confounders. An interesting result was that PP showed to be lower in hs-CRP > 3 mg/L group. There were few studies conducted the associations between hs-CRP and blood pressure. A cross-sectional study conducted among workers from the Colombian Oil Company showed DBP was higher for subjects in the second and fourth quartile of CRP groups [8]. Surapon et al found that hs-CRP concentration was significantly correlated with SBP and DBP but they did not study the confounders adjustment associations [30]. A further study is urgently needed to clarify the association between the hs-CRP and BP.

In this cross-sectional research of Yi people, the high prevalence of pre-hypertension and hypertension were observed, which were associated with the elevated hs-CRP. However, the correlation was unsustainable after adjusting for relevant confounders. It was demonstrated in a large number of studies that the levels of hs-CRP were up-regulated in the patients with pre-hypertension than that of the subjects with normal BPs [31, 32]. Nevertheless, hs-CRP could not be confirmed as the risk factor for hypertension by the above results. In fact, several epidemiological researches have indicated the unsustainability of the relationship between hs-CRP and BP. CARDIA has demonstrated that there was no significant association between hs-CRP and hypertension in perimenopausal (1.12 [0.99–1.27]) or postmenopausal (1.09 [0.95–1.26]) women [33]. The study published by Tanno–Sobetsu, as a prospective cohort study conducted in Japan, showed that there was no correlation between the high hs-CRP alone and the increased development of hypertension in both men and women [34]. However, most studies suggested that hs-CRP was positively associated with hypertension. A large cohort study followed for a median of 7.8 years revealed that the levels of hs-CRP played critical roles in the further development of hypertension, suggesting hypertension was part of inflammatory disorders [9]. Furthermore, it was demonstrated in another research followed for a median of 11 years that incident hypertension could be predicted by several independent factors including hs-CRP, abdominal obesity and smoking behavior [35]. It was also suggested that there was a remarkable correlation between CRP and incident hypertension after the normalization of other biomarkers [36]. Besides, BP was up-regulated by high levels of hs-CRP through inhibiting the nitric oxide production in endothelial cells [37, 38], resulting in vasoconstriction and evaluated production of endothelin 1 [39]. A meta-analysis of cohort studies represented that hs-CRP served as a risk indicator of the formation of hypertension, which also proven that stronger associations were existed in USA than in Asia (RRs: 1.73 vs. 1.03) [11], indicating that the regional differences in the effects of hs-CRP on hypertension should be taken into consideration. The regional differences may be explained by different ethnicities or races, which also led to various levels of hs-CRP [12–14]. It was also illustrated in a research conducted in South Africa among Caucasian and African women that the levels of hs-CRP was higher in African women (4.91 mg/L vs. 2.99 mg/L), which suggested the ethnic differences [40]. Additionally, for Yi people, hs-CRP and its associations with BPs were not obvious which provided relevant information.

There are still some limitations in this study. First, this is a cross-sectional study, and the results could not represent causal relations. Second, hs-CRP in this study was only assessed from one blood sample. But a trial indicated that the median hs-CRP concentration is close to the mean based on a cohort over 4 years [41]. Furthermore, although this sample is representative of the general population, some people with inflammatory diseases may be still included in the high hs-CRP group and further cohort studies should conducted to avoided this bias.

## Conclusions

In conclusion, this cross-sectional study shows that high hs-CRP is prevalent in Yi people, the effects of hs-CRP on SBP, DBP and MAP were relatively weak, however, compared with the hs-CRP < 1 mg/L group, the PP appeared to have lower values in hs-CRP > 3 mg/L group. This study does not support hs-CRP as a risk factor of prehypertension or hypertension. Further cohort studies should be conducted to clarify whether hs-CRP is a predictor of hypertension or not.

## Data Availability

The datasets used during the present study are available from the corresponding author upon reasonable request.

## Abbreviations

BMI: Body mass index
DBP: Diastolic blood pressure
MAP: Mean arterial pressure
PP: Pulse pressure
SBP: Systolic blood pressure
